# Bariatric Surgery in the United Kingdom: A Cohort Study of Weight Loss and Clinical Outcomes in Routine Clinical Care

**DOI:** 10.1371/journal.pmed.1001925

**Published:** 2015-12-22

**Authors:** Ian J. Douglas, Krishnan Bhaskaran, Rachel L. Batterham, Liam Smeeth

**Affiliations:** 1 Faculty of Epidemiology and Population Health, London School of Hygiene & Tropical Medicine, London, United Kingdom; 2 Centre for Obesity Research, Rayne Institute, Department of Medicine, University College London, London, United Kingdom; 3 University College London Hospitals Bariatric Centre for Weight Management and Metabolic Surgery, London, United Kingdom; 4 National Institute of Health Research, University College London Hospitals Biomedical Research Centre, London, United Kingdom; The George Institute for Global Health, The University of Sydney, AUSTRALIA

## Abstract

**Background:**

Bariatric surgery is becoming a more widespread treatment for obesity. Comprehensive evidence of the long-term effects of contemporary surgery on a broad range of clinical outcomes in large populations treated in routine clinical practice is lacking. The objective of this study was to measure the association between bariatric surgery, weight, body mass index, and obesity-related co-morbidities.

**Methods and Findings:**

This was an observational retrospective cohort study using data from the United Kingdom Clinical Practice Research Datalink. All 3,882 patients registered in the database and with bariatric surgery on or before 31 December 2014 were included and matched by propensity score to 3,882 obese patients without surgery. The main outcome measures were change in weight and body mass index over 4 y; incident diagnoses of type 2 diabetes mellitus (T2DM), hypertension, angina, myocardial infarction (MI), stroke, fractures, obstructive sleep apnoea, and cancer; mortality; and resolution of hypertension and T2DM. Weight measures were available for 3,847 patients between 1 and 4 mo, 2,884 patients between 5 and 12 mo, and 2,258 patients between 13 and 48 mo post-procedure. Bariatric surgery patients exhibited rapid weight loss for the first four postoperative months, at a rate of 4.98 kg/mo (95% CI 4.88–5.08). Slower weight loss was sustained to the end of 4 y. Gastric bypass (6.56 kg/mo) and sleeve gastrectomy (6.29 kg/mo) were associated with greater initial weight reduction than gastric banding (2.77 kg/mo). Protective hazard ratios (HRs) were detected for bariatric surgery for incident T2DM, 0.68 (95% CI 0.55–0.83); hypertension, 0.35 (95% CI 0.27–0.45); angina, 0.59 (95% CI 0.40–0.87);MI, 0.28 (95% CI 0.10–0.74); and obstructive sleep apnoea, 0.55 (95% CI 0.40–0.87). Strong associations were found between bariatric surgery and the resolution of T2DM, with a HR of 9.29 (95% CI 6.84–12.62), and between bariatric surgery and the resolution of hypertension, with a HR of 5.64 (95% CI 2.65–11.99). No association was detected between bariatric surgery and fractures, cancer, or stroke. Effect estimates for mortality found no protective association with bariatric surgery overall, with a HR of 0.97 (95% CI 0.66–1.43). The data used were recorded for the management of patients in primary care and may be subject to inaccuracy, which would tend to lead to underestimates of true relative effect sizes.

**Conclusions:**

Bariatric surgery as delivered in the UK healthcare system is associated with dramatic weight loss, sustained at least 4 y after surgery. This weight loss is accompanied by substantial improvements in pre-existing T2DM and hypertension, as well as a reduced risk of incident T2DM, hypertension, angina, MI, and obstructive sleep apnoea. Widening the availability of bariatric surgery could lead to substantial health benefits for many people who are morbidly obese.

## Abstract 2

In a UK cohort study, Ian Douglas and colleagues investigate weight, BMI, and related health outcomes after bariatric surgery.

## Editors' Summary

**Background:**

Obesity—having an unhealthy amount of body fat—is a growing threat to global public health. Worldwide, 13% of adults are obese, and, in the UK and the US, the statistics are even worse. A quarter and a third, respectively, of adults in these countries are obese. Obesity is defined as having a body mass index (BMI; an indicator of body fat calculated by dividing a person’s weight in kilograms by their height in meters squared) of ≥30 kg/m^2^. Compared to people with a healthy weight (a BMI of 18.5–24.9 kg/m^2^), overweight and obese people have an increased risk of developing type 2 diabetes, cardiovascular conditions such as hypertension (high blood pressure), myocardial infarction (heart attack), angina, and stroke, and they tend to die younger. People become overweight, and eventually obese, by consuming food and drink that contain more energy (calories) than they need for their daily activities. So, obesity can be prevented and reversed by eating less and exercising more.

**Why Was This Study Done?:**

People with severe obesity (BMI of 40 kg/m^2^ or more) who have tried but failed to control their weight through lifestyle changes sometimes undergo bariatric surgery (weight loss surgery). In the UK and the US, this approach is also recommended for obese individuals who have an obesity-related illness such as type 2 diabetes with a lower BMI of 35 kg/m^2^ or more. Techniques such as gastric band surgery, gastric bypass, and sleeve gastrectomy all lead to reduced energy intake, and in randomized controlled trials comparing bariatric surgery and lifestyle interventions, bariatric surgery is associated with greater weight loss. However, the results of clinical trials are not always replicated in routine clinical practice. Here, the researchers investigate whether there is an association between bariatric surgery and weight, BMI, and obesity-related co-morbidities (illnesses) in the UK by undertaking a retrospective cohort study (an observational study that compares recorded clinical outcomes in non-randomized groups of patients who received different treatments).

**What Did the Researchers Do and Find?:**

The researchers used the UK Clinical Practice Research Datalink, which contains anonymized clinical information about patients provided by general practitioners (primary care physicians), to identify 3,882 patients who had had bariatric surgery. They matched each patient (average BMI 44.7 kg/m^2^), according to the patient’s medications and constellation of risk factors, to an obese individual from the dataset who had not had bariatric surgery. This “propensity matching” technique is used in studies where patients are not allocated at random to receive a treatment, and is meant to control for confounding—the possibility that patients who receive the treatment may be otherwise distinct from patients who do not. According to this analysis, patients who had had bariatric surgery lost weight rapidly during the first four post-operative months (4.98 kg/month); their weight loss was sustained at a slower rate for up to four years. By contrast, there were no weight changes in the patients who did not have surgery. Notably, bariatric surgery was associated with a lower risk of type 2 diabetes onset, hypertension onset, angina onset, myocardial infarction, and obstructive sleep apnea (a sleep disorder) onset, and with the resolution of both type 2 diabetes and hypertension in those who already had these conditions when they underwent surgery. However, over an average of 3.4 years of follow-up, there was no evidence of any difference in the risk of death.

**What Do These Findings Mean?:**

These findings show that bariatric surgery delivered in routine clinical practice in the UK is associated with a substantial initial weight loss that is sustained for at least four years after surgery. They also show that bariatric surgery is associated with improvements in pre-existing type 2 diabetes and hypertension and with a reduced risk of developing several obesity-related co-morbidities. Because the data used in the study were recorded for patient management by primary care physicians, the researchers were unable to use strict diagnostic criteria for some outcomes, which may limit the accuracy of these findings. Nevertheless, these results suggest that widening the availability of bariatric surgery in the UK could provide substantial health benefits for many people who are morbidly obese. Indeed, the researchers calculate that, if the associations seen in this study are causal (an observational study cannot prove that a treatment causes a specific outcome), bariatric surgery could prevent and/or resolve many tens of thousands of cases of hypertension and type 2 diabetes and prevent similar numbers of cases of other obesity-related illnesses among the 1.4 million morbidly obese people living in the UK.

**Additional Information:**

This list of resources contains links that can be accessed when viewing the PDF on a device or via the online version of the article at http://dx.doi.org/10.1371/journal.pmed.1001925.

- The World Health Organization provides information on obesity (in several languages)
- The Institute for Health Metrics and Evaluation website provides the latest details about global obesity trends; the World Obesity Federation also provides information about the global obesity epidemic
- The UK National Health Service Choices website provides information about obesity (including some real stories), bariatric surgery (including some comments from patients), and healthy eating
- The US Centers for Disease Control and Prevention has information on all aspects of overweight and obesity
- ChooseMyPlate.gov is a resource provided by the US Department of Agriculture that provides individuals and healthcare professionals with user-friendly information on nutrition and physical exercise
- The US National Institute of Diabetes and Digestive and Kidney Diseases provides information on bariatric surgery and on weight control and healthy living
- MedlinePlus provides links to other sources of information on obesity and bariatric surgery (in English and Spanish)

## Introduction

The prevalence of obesity and related health problems is increasing [1]. Worldwide, over 600 million adults are obese, with a body mass index (BMI) of 30 kg/m^2^ or more [2]. Bariatric surgery is recommended for consideration in the United Kingdom and United States in people with a BMI of 40 kg/m^2^ or more and in people with a BMI of 35–40 kg/m^2^ plus additional obesity-related co-morbidities such as type 2 diabetes mellitus (T2DM), and for whom other weight control measures have failed. Bariatric surgery is also considered first line therapy in people with a BMI of 50 kg/m^2^ or more [3–5]. More recently, UK guidelines extended eligibility for bariatric surgery to include people with a BMI of 30–34.9 kg/m^2^ and recent T2DM [6].

Evidence for the effectiveness of bariatric surgery is primarily based on the results of randomised trials [7–10]. However, trials simultaneously measuring the effects of different surgery methods are scarce, and it is unclear how evidence from trials translates to population-based healthcare. As bariatric surgery is now being offered more to people with T2DM, the effectiveness of treatment in these patients needs to be better defined. We therefore used data from the UK Clinical Practice Research Datalink (CPRD) to characterise the association between bariatric surgery and weight, BMI, and a wide range of relevant clinical outcomes including diabetes, cardiovascular diseases (CVDs), fractures, cancer, and mortality.

## Methods

### Ethics

Scientific approval was obtained from the Medicines and Healthcare products Regulatory Agency’s Independent Scientific Advisory Committee, and ethical approval was granted by the London School of Hygiene & Tropical Medicine ethics committee.

### Clinical Practice Research Datalink

The CPRD contains anonymised information from UK general practitioners and includes ~8% of the UK population [11]. Information includes complete recording of consultations, diagnoses, prescribed medicines, and basic demographic data. The practices and patients are generally representative of the UK population [11], and data quality is subject to rigorous audits. The data have been used to conduct over 700 peer-reviewed published studies, and data validity has been shown to be high for a variety of diagnoses [12]. Measures of BMI are broadly representative, comparable with nationally representative UK survey data [13]. Data for this study were taken from all CPRD records to the end of December 2014. We originally intended to use linked data from Hospital Episode Statistics records to measure short-term outcomes following surgery and linked data from the Myocardial Ischaemia National Audit Project for ischaemic outcomes (see S3 Text), but insufficient linked data were available for this to be feasible.

### Overview of Methods

Selection of the analysis population was done in four stages summarised here and with further detail below. (1) We began by selecting patients with a record of undergoing bariatric surgery. (2) We then selected a pool of obese patients without surgery, matched to surgery patients by age, sex, general practice, and calendar period, with five non-surgery patients selected for each surgery patient. (3) Amongst these patients, we calculated a propensity score for receiving surgery based on a wide range of risk factors. (4) Each surgery patient was then matched 1:1 with the non-surgery patient with the closest propensity score. All further analyses were then conducted using the propensity-score-matched cohort. This staged process of population selection was used to avoid the need for calculating propensity scores for the entire CPRD population.

### Bariatric Surgery Group

Patients were included if they had a code indicating bariatric surgery during their registration period in the CPRD (see S1 Text for code list) and had at least 12 mo of prior registration in the CPRD before the date of the surgery, to ensure that any outcomes identified reflected incident events and not retrospective recording [14]. Patients were excluded if they previously had a record indicating reversal of bariatric surgery (e.g., gastric band removal).

### Eligible Comparison Group

A base comparison group was initially identified from the CPRD comprising individuals with at least one measure of BMI ≥ 40 kg/m^2^ at any point in their registration, in order to define a group of people who may have been eligible for bariatric surgery. The maximum follow-up duration for patients was 15 y, and non-surgery patients remained eligible if they also had measures of BMI below 40 kg/m^2^ recorded during their follow-up. The non-surgery group was therefore not restricted to patients for whom all measures of BMI were ≥40 kg/m^2^. All eligible bariatric surgery patients were then matched with up to five of these individuals, matching on age (within 2.5 y), sex, general practice, and presence in the CPRD on the date bariatric surgery was recorded (termed the index date). Non-surgery patients had to have no record of bariatric surgery before this date and at least 12 mo of prior registration in the database.

### Outcomes

The outcomes were change in weight and BMI; incident diagnoses of T2DM, hypertension, angina, myocardial infarction (MI), stroke, fractures, obstructive sleep apnoea, and cancer; mortality; and resolution of hypertension and T2DM.

Height and weight records were extracted from clinical files. Implausible records for obese adults were discarded (any weight <40 kg or >300 kg; <1% all recorded weight measures). Weights between 225 and 300 kg were discarded if other measures on the same day were <225 kg or where the ratio to other recorded weights for the individual was >1.5 (<0.01% of all recorded weight measures). For patients with recorded height, BMI was calculated for each weight record, and the nearest weight prior to the index date was taken as the baseline weight.

For clinical outcomes, Read codes were used to identify records indicating incident events. Hypertension and T2DM resolution were defined by either a code stating resolution or the withdrawal of drug treatment for the condition for at least a 6-mo period.

### Covariates

CPRD records were searched for records of T2DM, hypertension, coronary heart disease, cerebrovascular disease, peripheral vascular disease, other atheroma, smoking status, alcohol consumption, and use of statins, oral antidiabetic drugs (OAD), and insulin.

### Propensity Score Matching

For all bariatric surgery patients and their matches, a conditional logistic regression model was constructed with bariatric surgery as the outcome, and status regarding the following factors defined prior to the index date as covariates: T2DM, hypertension, coronary heart disease, cerebrovascular disease, peripheral vascular disease, other atheroma, smoking status, alcohol consumption, and use of insulin, OADs, and statins. For variables with missing data (smoking and alcohol consumption), a missing category was used in the analysis, since multiple imputation cannot be used in conjunction with propensity scores. Propensity scores were then calculated, and each surgery patient was matched 1:1 to the person without surgery with the closest propensity score, choosing matches at random where more than one possible match had the same score. All further analyses were done using this propensity-score-matched cohort. Standardised differences (differences in means or proportions divided by standard error) were calculated for each baseline variable to determine any imbalances [15], with differences >0.2 suggesting imbalance.

### Statistical Analysis

#### Weight and BMI changes

Non-surgery patients tended not to have a recent weight measure on their matched index date. To avoid errors associated with using out-dated measures [13], follow-up for this group was started at the weight measured nearest to the index date and ended at the earliest of 4 y later, death, bariatric surgery, first orlistat or sibutramine prescription, transfer from practice, or last data recording date. Change in weight and BMI after bariatric surgery was modelled using mixed effects linear regression.

The rate of weight loss was anticipated to change with time since surgery, and so a linear spline model was fitted to allow the calculation of an approximate rate of change in each distinct phase of weight loss. The Akaike information criterion was used to determine the optimal number and time point of spline knots. A restricted cubic spline model was later fitted after advice from peer review, to avoid sharp discontinuity in rate measures, and both models are presented. Right censoring was at the last recorded weight within 4 y of the index date. From the final linear spline model, weight and BMI change and their 95% confidence intervals for the study population were estimated over 4 y. Separate analyses were conducted in patients with T2DM or CVD because of the specific importance of weight reduction therapies among these groups [5]. The weight and BMI change associated with each surgical procedure was measured separately where patient numbers permitted. For each surgery type, we compared baseline BMI and used ANOVA to determine any differences. Post hoc we also assessed the weight and BMI change for each surgery type separately in patients with T2DM, given recent changes to UK guidelines.

To determine whether changes in weight over time were biased by preferentially observing later measures for patients whose baseline weight systematically differed from the group as a whole, the baseline weight of patients contributing measures beyond 4 and 12 mo was compared with the baseline weight of all patients.

#### Clinical outcomes

For clinical outcomes, Cox regression was used to determine the hazard ratio (HR) for each event. For all analyses, the highest and lowest 5% propensity score bands were excluded (trimming) since patients treated contrary to extreme scores can introduce bias if important information about their health status is missing [16]. A sensitivity analysis was done without trimming. For each analysis, all individuals with a history of the specific outcome were excluded. We ensured that the proportional hazards assumption was met for all analyses. Bariatric surgery patients were compared with propensity-score-matched non-surgery individuals, with no statistical adjustments made, starting follow-up at the index date. Right censoring was applied using the earliest of these events: the outcome of interest, death, transfer away from the practice, or the last data collection date for the practice. For resolution of T2DM and hypertension, analyses were restricted to individuals with a history of each condition at the index date. Secondary analyses assessed the association between bariatric surgery and each outcome in patients with either T2DM or any CVD, and for each subtype of bariatric surgery where numbers permitted. Following peer review, a separate analysis of mortality within 30 d of surgery was also conducted, to assess any association between bariatric surgery and short-term mortality (to investigate peri-operative mortality). Initially, incident fatty liver was also included as an outcome, but following peer review, this was removed because of a lack of reliable diagnostic information on the more important outcome of non-alcoholic steatohepatitis.

Post hoc we explored the results seen for T2DM resolution by examining HbA1c levels, where recorded. After finding no protective association between bariatric surgery and mortality, we looked separately at the first year of follow-up and the remainder, since others have reported delayed protective associations [17].

Health Survey for England estimates that currently 1.4 million residents in England are morbidly obese (BMI ≥ 40 kg/m^2^) [18]. For all outcomes where an association with surgery was detected, we estimated the absolute number of outcomes that surgery could potentially prevent, by applying the relative risks obtained in our analysis to the rate of events detected in the non-surgery group, and scaling to 1.4 million people.

All analyses were conducted using Stata 13.0 (StataCorp).

## Results

**Fig 1:**
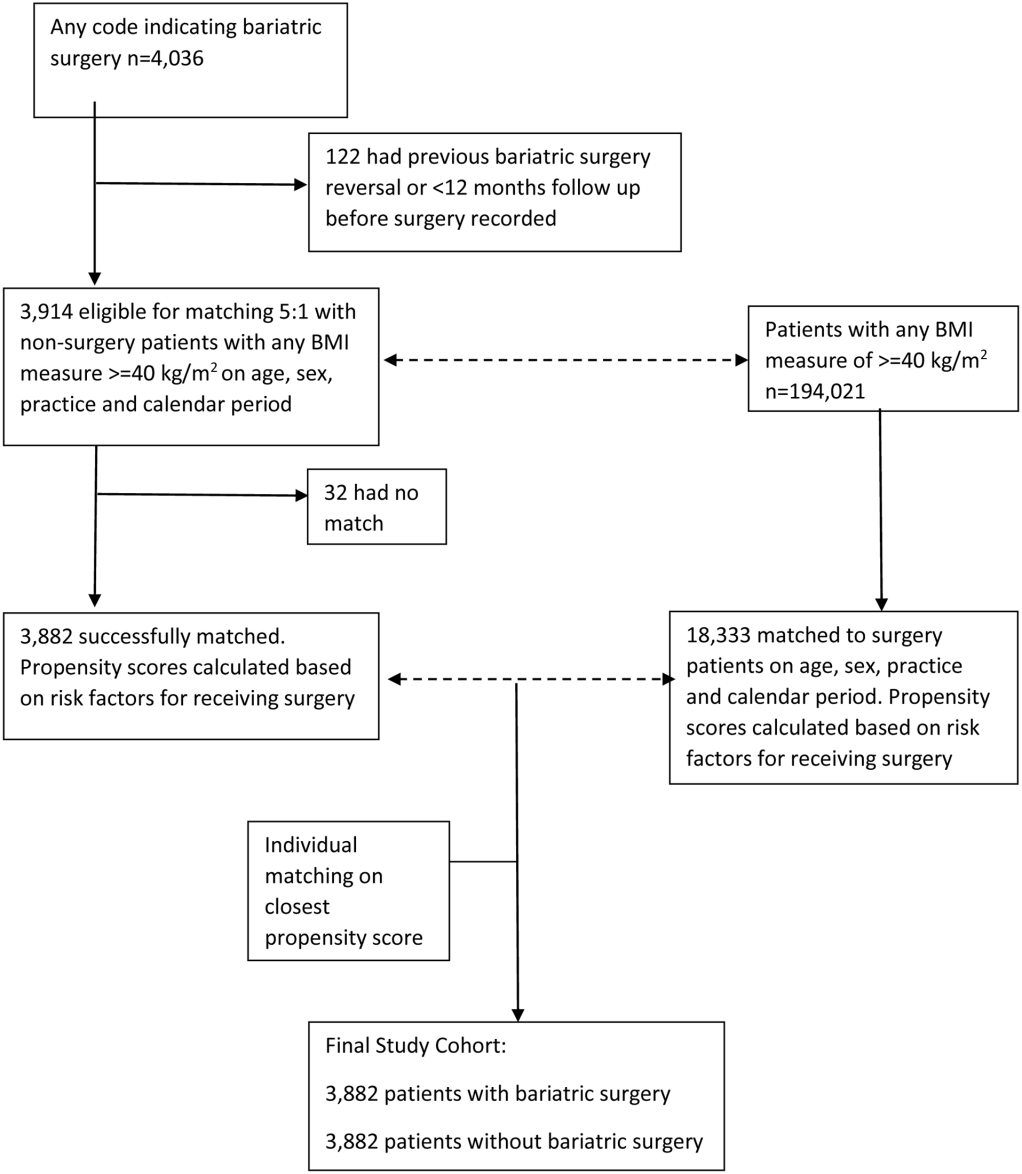
Flow diagram of study population.

A total of 4,036 patients were identified with a record of bariatric surgery (Fig 1). After applying exclusion criteria, 3,914 remained. Of these, 3,882 were matched on age, sex, practice, and calendar period to 18,333 patients without bariatric surgery from a total population of 194,021 people with any single BMI measure of ≥40 kg/m^2^. No matches were identified for 32 patients. Propensity scores were then calculated for the age-, sex-, practice-, and calendar-period-matched patients. Following this, each surgery patient was matched to the patient without surgery with the closest propensity score. The distribution of baseline characteristics for this final matched cohort is shown in Table 1. The mean age was 45 y, and 81% were women, with mean follow-up of 3.4 y. There were 1,425 surgery patients who were followed for more than 4 y (maximum 14 y). Standardised differences show that the distribution of all characteristics was very similar between the groups, with the exception of BMI. Nearly all (99.5%) of the bariatric surgeries performed were gastric band, gastric bypass, or sleeve gastrectomy. Bariatric surgery patients had a mean BMI of 44.7 kg/m^2^, compared with 42.1 kg/m^2^ amongst patients without surgery. In the non-surgery group, 56% had a record of receiving a lifestyle intervention for weight loss, most frequently advice about food intake (40%). However, the absence of a record of receiving a lifestyle intervention should not be interpreted as a lack of intervention as it is likely such advice is not well recorded.

**Table 1:** Background characteristics of propensity-score-matched cohort of patients with and without bariatric surgery.

Characteristic	Bariatric Surgery Group (*n =* 3,882)	No Surgery Group (*n =* 3,882)	Standardised Difference
**Age (y), mean (SD)**	45 (11)	45 (11)	0.012
**Female, *n* (percent)**	3,126 (80.5%)	3,166 (81.6%)	−0.026
**Years of follow-up after index date, mean (SD)**	3.4 (2.3)	3.4 (2.4)	0.013
**Smoking, *n* (percent)**			
Non-smoker	1,724 (44.4%)	1,725 (44.4%)	−0.005
Current smoker	564 (14.5%)	532 (13.7%)	0.025
Ex-smoker	1,592 (41.0%)	1,623 (41.8%)	−0.013
Missing	2 (0.1%)	2 (0.1%)	−0.013
**Alcohol consumption, *n* (percent)**			
Non-drinker	628 (16.2%)	608 (15.7%)	0.016
Ex-drinker	371 (9.6%)	349 (9.0%)	0.019
Current drinker: amount not known	21 (0.5%)	21 (0.5%)	0.000
Current drinker: <2 units/d	984 (25.4%)	956 (24.6%)	0.017
Current drinker: 3–6 units/d	1,278 (32.9%)	1,351 (34.8%)	−0.042
Current drinker: >6 units/d	262 (6.8%)	256 (6.6%)	0.008
Missing	338 (8.7%)	341 (8.8%)	−0.003
**Last BMI (kg/m** ^**2**^ **) before index date**			
Mean (SD)	44.7 (8.8)	42.1 (6.5)	0.342
Number (percent) missing	59 (1.5%)	67 (1.7%)	
Lag to index date (d), mean (SD)	278 (558)	539 (829)	
**Co-morbidities and drugs used, *n* (percent)**			
CVD	53 (1.4%)	40 (1.0%)	0.031
Coronary heart disease	145 (3.7%)	135 (3.5%)	0.015
Peripheral vascular disease	24 (0.6%)	27 (0.7%)	−0.007
Other atheroma	* (0%)	* (0%)	0.013
Hypertension	1,313 (33.8%)	1,323 (34.1%)	−0.004
T2DM: diagnosed or treated	1,320 (34.0%)	1,296 (33.4%)	0.012
T2DM: OAD treatment	824 (21.2%)	686 (17.7%)	0.090
T2DM: insulin treatment	252 (6.5%)	256 (6.6%)	−0.003
Statin use	1,015 (26.2%)	987 (25.4%)	0.017
**Type of bariatric surgery, *n* (percent)**			
Gastric band	1,829 (47.1%)		
Gastric bypass	1,421 (36.6%)		
Sleeve gastrectomy	613 (15.8%)		
Duodenal switch	* (0.1%)		
Gastric stapling	6 (0.2%)		
Stomach partition (not elsewhere classified)	5 (0.1%)		
Mason vertical banded gastroplasty	* (0.1%)		
**Lifestyle intervention for non-surgery group, *n* (percent)**			
Any intervention		2,153 (56%)	
Diet advice		1,535 (40%)	
Seen by diet specialist		800 (21%)	
Activity/exercise advice		793 (20%)	
Non-specific		420 (11%)	

*Counts <5 cannot be given precisely due to anonymity considerations.

SD, standard deviation.

### Change in Weight and BMI

**Fig 2:**
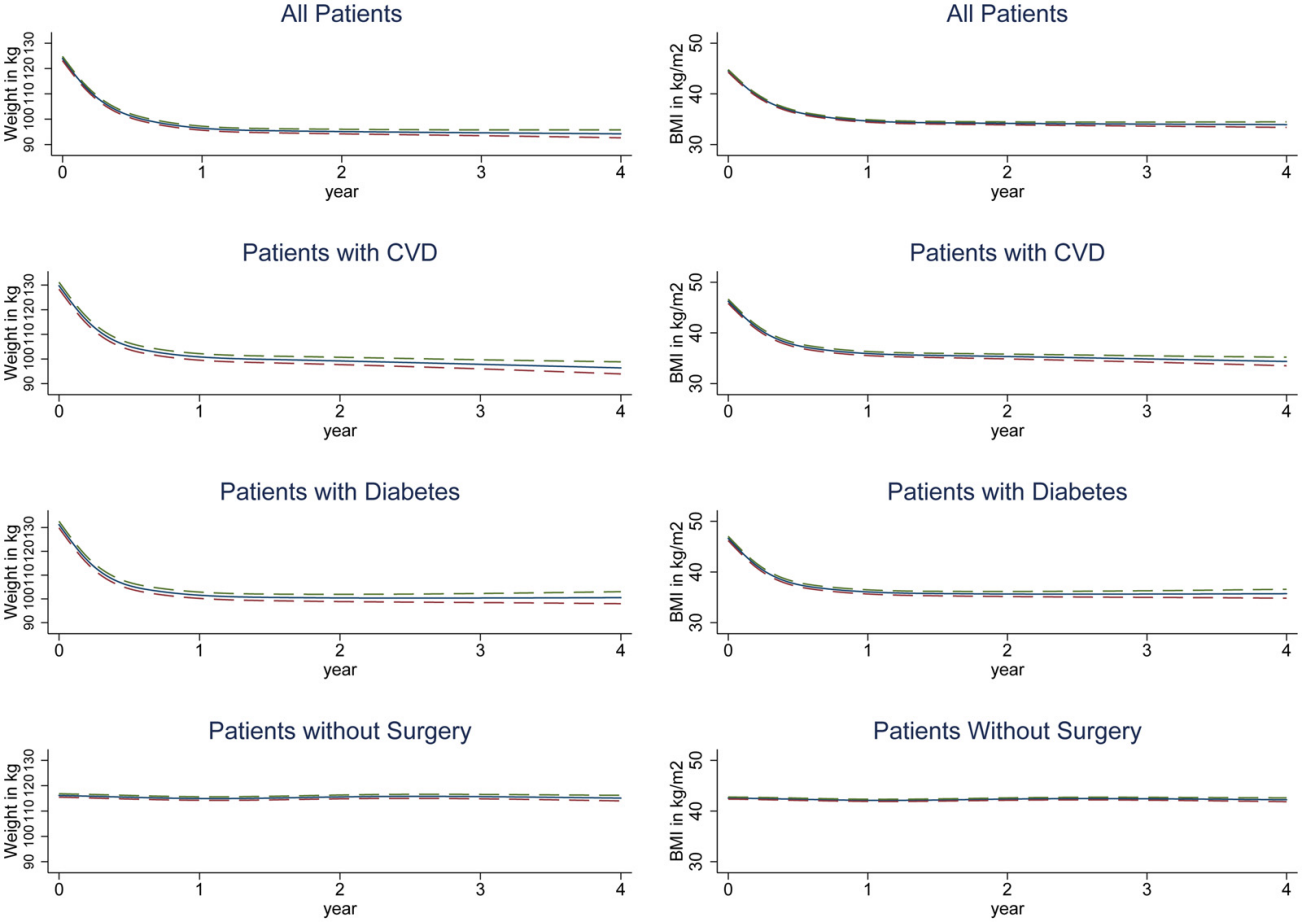
Estimated mean change in weight and BMI over 4 y following bariatric surgery or no surgery. Dashed lines show 95% CIs.

For all weight and BMI analyses, figures show the results obtained from restricted cubic spline models and tables show the results from linear spline models. Amongst surgery patients, the 4 mo following surgery saw rapid weight loss, with a mean rate of 4.98 kg/mo (95% CI 4.88–5.08) (Fig 2; Table 2). Slower weight loss continued for the remaining follow-up. When stratified by surgery type (Fig 2; Table 2), gastric bypass was associated with the largest initial weight reduction rate, 6.56 kg/mo in the first 4 mo. Patients with sleeve gastrectomy had similar results, with a weight reduction rate of 6.29 kg/mo, while gastric band was associated with a less dramatic reduction of 2.77 kg/mo. There was no evidence of weight gain to the end of 4 y in any group. People with CVD or T2DM had more rapid initial weight loss, contrary to expectations [19] (Fig 2; Table 2), so we stratified the data by surgery type in patients with T2DM and CVD. Amongst people with T2DM, gastric bypass was more prevalent than in the wider group (49% versus 36%), as was sleeve gastrectomy (19% versus 16%), with fewer undergoing gastric band (27% versus 47%). When stratified by surgery type, the results in people with T2DM were largely similar to those seen in the group as a whole, with the exception of patients who underwent sleeve gastrectomy: these patients gained an average of 0.21 kg/mo from 13 mo onwards (S1 Table). Similarly, patients with CVD were more likely to have gastric bypass (44%), with fewer having gastric bands (39%) or sleeve gastrectomy (15%); however, there were insufficient patients with CVD to perform stratified analyses.

**Table 2:** Rate of change in weight and BMI over 3-y follow-up.

Intervention and Follow-Up Period	*N* at Follow-Up Period Start	*N* with Weight Measure	Estimated Weight Change, kg/mo (95% CI)	Estimated BMI Change, kg/m^2^/mo (95% CI)
**Bariatric surgery (any form)**				
1–4 mo	3,882	3,847	−4.98 (−5.08 to −4.88)	−1.78 (−1.82 to −1.75)
5–12 mo	3,372	2,884	−1.01 (−1.08 to −0.96)	−0.37 (−0.39 to −0.35)
13–48 mo	3,020	2,258	−0.04 (−0.08 to 0.00)	−0.01 (−0.03 to 0.00)
**Gastric band**				
1–4 mo	1,881	1,828	−2.77 (−2.92 to −2.62)	−1.00 (−1.04 to −0.94)
5–12 mo	1,758	1,165	−0.84 (−0.93 to −0.76)	−0.30 (−0.33 to −0.27)
13–48 mo	1,626	961	−0.05 (−0.09 to 0.00)	−0.02 (−0.03 to 0.00)
**Gastric bypass**				
1–4 mo	1,535	1,489	−6.56 (−6.69 to −6.42)	−2.36 (−2.40 to −2.31)
5–12 mo	1,368	1,172	−1.50 (−1.59 to −1.42)	−0.55 (−0.58 to −0.52)
13–48 mo	1,201	890	0.02 (−0.04 to 0.08)	0.01 (−0.01 to 0.03)
**Sleeve gastrectomy**				
1–4 mo	663	639	−6.29 (−6.55 to −6.04)	−2.21 (−2.29 to −2.12)
5–12 mo	563	428	−0.97 (−1.13 to −0.80)	−0.35 (−0.41 to −0.30)
13–48 mo	480	299	0.05 (−0.06 to 0.17)	0.03 (−0.01 to 0.06)
**Bariatic surgery in patients with CVD**				
1–4 mo	1,396	1,368	−5.40 (−5.54 to −5.25)	−1.92 (−1.97 to −1.87)
5–12 mo	1,278	1,126	−0.96 (−1.05 to −0.87)	−0.35 (−0.38 to −0.32)
13–48 mo	1,148	911	−0.09 (−0.14 to −0.03)	−0.03 (−0.05 to −0.01)
**Bariatric surgery in patients with T2DM**				
1–4 mo	1,338	1,294	−5.65 (−5.79 to −5.50)	−2.00 (−2.05 to −1.95)
5–12 mo	1,220	1,094	−0.98 (−1.07 to −0.89)	−0.35 (−0.38 to −0.32)
13–48 mo	1,084	866	0.00 (−0.05 to 0.06)	0.00 (−0.02 to 0.02)
**No intervention**				
1–12 mo	3,882	3,877	−0.11 (−0.14 to −0.08)	−0.04 (−0.05 to −0.03)
13–24 mo	3,592	2,044	0.07 (0.03 to 0.12)	0.03 (0.01 to 0.04)
25–48 mo	3,176	1,510	−0.01 (−0.04 to 0.02)	0.00 (−0.01 to 0.01)

The mean baseline BMI for each surgery type was as follows: gastric band, 42.3 kg/m^2^; gastric bypass, 46.9 kg/m^2^; and sleeve gastrectomy, 47.1 kg/m^2^. Using ANOVA, there was evidence that these differences were more than expected by chance (*p ≤* 0.001). As expected, the pattern for BMI during follow-up closely followed that for weight in all analyses (Fig 2; Table 2).

The mean weight of surgery patients at baseline was 124 kg (standard deviation [SD] = 28 kg). For those contributing weight measures past 4 mo (*n =* 2,152) and 12 mo (*n =* 1,604), mean baseline weight was 127 kg (SD = 28 kg) and 128 kg (SD = 28 kg), respectively, indicating that patients with later weight measures were representative of the whole group in terms of baseline weight.

Amongst the group without bariatric surgery, no overall changes in weight or BMI were detected over the 4-y period.

### Clinical Outcomes

Strong protective associations were detected between bariatric surgery and T2DM onset (HR = 0.68, 95% CI 0.55–0.83), first treatment with an OAD (HR = 0.26, 95% CI 0.18–0.37), first treatment with insulin (HR = 0.22, 95% CI 0.11–0.43), hypertension onset (HR = 0.35, 95% CI 0.27–0.45), angina onset (HR = 0.59, 95% CI 0.40–0.87), MI (HR = 0.28, 95% CI 0.10–0.74), and obstructive sleep apnoea onset (HR = 0.55, 95% CI 0.37–0.82) (see Table 3). We also found strong associations between bariatric surgery and the resolution of T2DM (HR = 9.29, 95% CI 6.84–12.62) and hypertension (HR = 5.64, 95% CI 2.65–11.99). HbA1c measures were evaluated post hoc for patients discontinuing diabetes treatment. Measures were available for 236 (74%) surgery patients: for 144 (45%) patients, all subsequent measures were <6.0%, while 92 (29%) patients had at least one measure ≥ 6.0% after the estimated date of resolution. No association was seen between bariatric surgery and stroke, fractures, cancer, or mortality. The sensitivity analysis with all patients with extreme propensity scores included had very similar results (S2 Table). When stratified by surgery type, the pattern with respect to outcomes was similar for each type, with the exception of the outcomes T2DM and hypertension resolution, where stronger associations were seen for gastric bypass and sleeve gastrectomy than for gastric banding (S3 Table). The post hoc analysis for mortality, stratified on follow-up period, found a HR of 1.10 (95% CI 0.59–2.06) for the first year after surgery and 0.77 (95% CI 0.48–1.24) after the first year. In the analysis of mortality within 30 d of surgery, fewer than five surgery patients (0.08%) died, compared with no deaths in the non-surgical group (because of CPRD restrictions around patient anonymity, counts of less than five cannot be given precisely).

**Table 3:** Association between bariatric surgery and health outcomes.

Outcome	Group	*N* *	Number (Percent) with Outcome	Median Follow-Up (y)	HR (95% CI)	*p-*Value
**T2DM**						
First diagnosis	No surgery	2,552	237 (6.6%)	2.9	—	
	Surgery	2,397	158 (9.3%)	3.1	0.68 (0.55–0.83)	<0.001
First OAD	No surgery	2,891	149 (4.7%)	2.9	—	
	Surgery	3,141	37 (1.3%)	3.2	0.26 (0.18–0.37)	<0.001
First insulin	No surgery	3,463	47 (1.3%)	3.0	—	
	Surgery	3,514	10 (0.3%)	3.1	0.22 (0.11–0.43)	<0.001
Resolution	No surgery	1,175	47 (4.0%)	2.6	—	
	Surgery	1,113	321 (28.8%)	1.8	9.29 (6.84–12.62)	<0.001
**Hypertension**						
Diagnosis	No surgery	2,498	219 (8.8%)	2.8	—	
	Surgery	2,444	79 (3.2%)	2.9	0.35 (0.27–0.45)	<0.001
Resolution	No surgery	1,256	8 (0.6%)	2.8	—	
	Surgery	1,255	46 (3.7%)	2.8	5.64 (2.65–11.99)	<0.001
**Angina**	No surgery	3,565	68 (1.9%)	3.0	—	
	Surgery	3,463	40 (1.2%)	3.0	0.59 (0.40–0.87)	0.007
**MI**	No surgery	3,732	18 (0.5%)	3.0	—	
	Surgery	3,618	5 (0.1%)	3.1	0.28 (0.10–0.74)	0.01
**Stroke**	No surgery	3,748	19 (0.5%)	3.0	—	
	Surgery	3,683	17 (0.5%)	3.0	0.91 (0.47–1.76)	0.86
**Fractures**						
Hip	No surgery	3,749	7 (0.2%)	3.0	—	
	Surgery	3,686	8 (0.2%)	3.1	1.15 (0.42–3.18)	0.78
Wrist	No surgery	3,566	18 (0.5%)	3.0	—	
	Surgery	3,486	27 (0.8%)	3.0	1.56 (0.86–2.84)	0.14
Spine	No surgery	3,753	11 (0.3%)	3.0	—	
	Surgery	3,694	16 (0.4%)	3.0	1.50 (0.69–3.23)	0.30
Any	No surgery	3,535	32 (0.9%)	3.0	—	
	Surgery	3,447	39 (1.1%)	3.0	1.26 (0.79–2.01)	0.34
**Obstructive sleep apnoea**	No surgery	3,637	71 (2.0%)	3.0	—	
	Surgery	3,248	36 (1.1%)	3.1	0.55 (0.37–0.82)	0.004
**Any cancer**	No surgery	3,536	138 (3.9%)	2.9	—	
	Surgery	3,452	127 (3.7%)	3.0	0.94 (0.74–1.20)	0.64
**Mortality**	No surgery	3,774	50 (1.4%)	3.0	—	
	Surgery	3,714	53 (1.4%)	3.1	0.97 (0.66–1.43)	0.87

*Each analysis population consisted of patients without the condition of interest at the start of follow-up.

### Absolute Effects

Assuming the associations we report are causal, the number of events that bariatric surgery could have prevented amongst the ~1.4 million morbidly obese people in England over the same period as this study is as follows: hypertension, *n =* 79,780; angina, *n =* 10,949; T2DM, *n =* 41,600; MI, *n =* 4,861; and obstructive sleep apnoea, *n =* 12,299. Furthermore, disease resolution might have occurred in 107,807 people with T2DM and 13,464 people with hypertension.

## Discussion

We assessed outcomes following bariatric surgery for obesity in routine clinical practice among the general UK population. Amongst patients with an average BMI of 44.7 kg/m^2^, dramatic reductions in weight and BMI were sustained over a 4-y period, with gastric bypass and sleeve gastrectomy associated with greater weight loss than gastric band. Estimated 4-y weight loss was 38 kg for gastric bypass, 31 kg for sleeve gastrectomy, and 20 kg for gastric band.

We found evidence that surgery has major beneficial associations with several clinical outcomes, with reductions in risk seen for incident T2DM, hypertension, angina, MI, and obstructive sleep apnoea. Resolution of T2DM and hypertension was also seen. Crude estimates suggest that large numbers of obese people could avoid future harmful clinical outcomes if they were offered surgery. Although we acknowledge that not all patients are suitable for bariatric surgery, it appears that better access to bariatric surgery, where appropriate, could lead to a considerable reduction in the burden of disease and substantial cost savings for the health system. Notably, only 5,558 bariatric procedures were done in the UK in 2013, compared with 37,300 in France, where the prevalence of obesity is lower than in the UK [20]. Similarly, whilst 154,276 bariatric procedures were performed in the US and Canada in 2013, an estimated 6.4% of the adult US population has a BMI ≥ 40 kg/m^2^, equating to around 14 million people, many of whom could likely benefit from surgery [21].

On the whole, the outcomes seen following surgery were similar in people with T2DM. There was a suggestion that people with T2DM undergoing sleeve gastrectomy may start to regain weight a year after surgery. However, this was a post hoc analysis and would need to be investigated further in other populations.

Resolution of T2DM and hypertension was more common in people receiving gastric bypass or sleeve gastrectomy than in those undergoing gastric band surgery. For other outcomes, there was little evidence of differential associations by surgery type.

### Strengths and Weaknesses

This is one of the largest population-based studies examining a wide range of outcomes following bariatric surgery. The data are representative of the UK population and reflect current medical practice, and so the results are likely to generalise to the UK population as a whole. Patients are not lost to follow-up in the CPRD; they remain registered with their practice unless they transfer to another, at which point their follow-up is censored. This occurred in only ~10% of the patients included here, reducing the potential for biased results due to selective loss to follow-up.

BMI recorded in the CPRD has good concordance with the Health Survey for England at the population level when using BMI measured within 3 y [13]. For our study, measures were applied on the date taken, except baseline weight for the surgery group, where the most recent pre-surgery measure was used. The average time from measurement to surgery was less than 1 y, and we therefore do not anticipate major bias. Although weight measures were not available for all patients after surgery, the majority of patients remaining in follow-up did have valid measures that could be included in our analyses. Patients contributing weight measures were similar to those without measures, suggesting there were no systematic differences determining whether weight was recorded.

Our case definition for T2DM and hypertension resolution was based on discontinuation of pharmacological treatment because measures of HbA1c and blood pressure were not routinely recorded for all patients. It is possible that these outcomes were subject to some inaccuracy, which would tend to lead to underestimation of any change in risk following surgery.

At baseline, average BMI was lower in the non-surgery group, and some in this group had a BMI below 40 during follow-up. This may have led to underestimation of relative effect sizes if this reduction in BMI conferred a general protective effect on the non-surgery group, although the groups were well matched on other co-morbidities. Mean baseline BMI was also a little lower in people undergoing gastric banding than in those undergoing gastric bypass or sleeve gastrectomy, and so caution is needed when interpreting potentially different results across surgery types.

We were unable to look in detail at short-term adverse outcomes associated with surgery, other than short-term mortality, which was very low (0.08%). However, the UK National Bariatric Surgery Registry recently reported detailed short-term adverse outcomes from 18,283 bariatric surgery procedures conducted in the UK and Ireland between 2011 and 2013, complementing the longer term outcomes we report here [22] and based largely on the same underlying population. They reported an in-hospital mortality rate of 0.07%, similar to our findings. The average post-operative hospital stay was 2.7 d, with only 3% remaining in hospital longer than 5 d. The surgical complication rate for primary operations was 2.9%, with the most common complications cited as vomiting/poor intake, fluid/electrolyte problems, and pneumonia and other infections. Cardiovascular complications were rare (0.3%). Procedure-related complications, e.g., band slippage, bleeds, and obstructions, were reported in 2.4% of patients, and revision surgery was required in 1.4% of cases. Revision surgery was most common for gastric bands (1.8%) and was rarer for gastric bypass (0.4%) and sleeve gastrectomy (0.4%). There was little difference between the procedures for other factors. Most other studies have suggested that sleeve gastrectomy may be safer than gastric bypass [23–27], though some found no relevant differences [28]. These findings suggest that adverse outcomes after bariatric surgery are rare, hospital stays are short, and the benefits we report here outweigh the short-term risks involved.

### Comparison with Other Studies

A recent meta-analysis of randomised trials comparing surgery against non-surgical interventions found that an additional 26 kg was lost by surgery patients, but highlighted that evidence beyond 2 y was lacking [29]. Estimates of absolute weight lost in trials of gastric bypass range from to 29.4 to 50.6 kg over 2 y [9,10,30,31]. For gastric band, estimates range from 17 to 21.1 kg [32,33], and for sleeve gastrectomy, from 25.1 to 29.5 kg [18,21]. Picot et al. [34] compared evidence for different surgery types and concluded that gastric bypass was more effective than gastric band. Evidence for differences between gastric bypass and sleeve gastrectomy is less clear [17,35–37].

A US observational cohort reported a 3-y weight reduction of 20 kg for gastric band patients and 41 kg for gastric bypass patients [38]. Although longer term follow-up is available from the Swedish Obese Subjects (SOS) study, direct comparison is difficult as the majority underwent vertical banded gastroplasty, which was rarely used in our study [39].

Our results are similar to those in the studies described above. Moreover, our findings are remarkably similar to the results reported in the US cohort [38], demonstrating the strength of routine primary care records for measuring outcomes following bariatric surgery.

For other clinical outcomes, our findings are largely in line with the results of other studies, though we did not find the strong protective association between bariatric surgery and mortality seen by others [17], possibly because of the lack of longer term data in our study. Notably, the results of our post hoc analysis were consistent with a survival benefit a year after surgery—similar to the long-term protective effect seen in the SOS study [34]—but this survival benefit did not reach statistical significance. The effect estimates we report for resolution of T2DM and hypertension are slightly weaker than those seen in some studies, but are consistent with effects seen in the SOS study, and it is clear that this effect size has varied in other studies depending on the case definition used [39–41]. Few studies have reported on vascular outcomes, with most reporting associations of bariatric surgery with vascular risk factors instead [42]. Our results suggest a slightly stronger protective association between surgery and MI than reported in the SOS study [43], though the mix of surgery subtypes varies considerably between our studies and could explain these differences. Notably, we found no association between bariatric surgery and the risk of stroke.

Similar to Booth et al. [44], who also used data from the CPRD, we found a protective association between bariatric surgery and T2DM onset, although the HR of 0.68 we detected was weaker than the HR of 0.2 previously reported. It is possible that this difference reflects a difference in the case definition used for T2DM, but, notably, we confirm a similarly strong protective association between surgery and first OAD treatment for T2DM, with a HR of 0.28.

Few studies have accurately measured the effects of surgery on cancer, with the exception of the SOS study, which found a strong protective effect in women but not men [45]. We found no such association here, although it is possible we had insufficient follow-up to detect any genuinely causal effect, which may take many years to accrue, or that any benefits are cancer-site-specific [46].

Longer term measures of outcomes following surgery are needed. This will require several years’ more data to accrue, especially for gastric bypass and sleeve gastrectomy.

## Conclusion

Bariatric surgery as delivered in the UK healthcare system leads to dramatic weight loss, generally sustained at least 4 y after surgery. This weight loss is accompanied by substantial improvements in pre-existing T2DM and hypertension as well as a reduced risk of incident T2DM, hypertension, angina, MI, and obstructive sleep apnoea. Our results also suggest possible differences in outcomes following specific types of bariatric surgery amongst people with T2DM that need to be investigated in other populations.

## Supporting Information

S1 Table Rate of change in weight following surgery in people with type 2 diabetes stratified by surgery type. (DOCX)

S2 Table Association between bariatric surgery and health outcomes without trimming patients with extreme propensity scores. (DOCX)

S3 Table Association between bariatric surgery and health outcomes stratified by type of surgery, baseline type 2 diabetes, and baseline CVD. (DOCX)

S1 Text CPRD code list for bariatric surgery. (DOCX)

S2 Text STROBE checklist. (DOC)

S3 Text Study protocol as approved by the Medicines and Healthcare Products Regulatory Agency’s Independent Scientific Advisory Committee. (DOC)

## Abbreviations

BMI: body mass index
CPRD: Clinical Practice Research Datalink
CVD: cardiovascular disease
HR: hazard ratio
MI: myocardial infarction
OAD: oral antidiabetic drug
SD: standard deviation
SOS study: Swedish Obese Subjects study
T2DM: type 2 diabetes mellitus

## References

[1] Department of Health. Obesity general information. 1 February 2011. Available: http://webarchive.nationalarchives.gov.uk/+/www.dh.gov.uk/en/Publichealth/Obesity/DH_078098. Accessed 8 May 2015.

[2] World Health Organization. Obesity and overweight. Fact sheet no. 311. January 2015. Available: http://www.who.int/mediacentre/factsheets/fs311/en/index.html. Accessed 8 May 2015.

[3] National Institute for Health and Care Excellence. Obesity prevention. NICE guidelines CG43. December 2006. Available: http://guidance.nice.org.uk/CG43. Accessed 8 May 2015.

[4] National Health Service Commissioning Board. Clinical commissioning policy: complex and specialised obesity surgery. April 2013. NHSCB/A05/P/a. Available: https://www.england.nhs.uk/wp-content/uploads/2013/04/a05-p-a.pdf. Accessed 20 November 2015.

[5] National Heart, Lung, and Blood Institute Obesity Education Initiative Expert Panel on the Identification, Evaluation, and Treatment of Obesity in Adults. Clinical guidelines on the identification, evaluation, and treatment of overweight and obesity in adults: the evidence report. Report No. 98–4083. Bethesda (Maryland): National Heart, Lung, and Blood Institute; 1998.

[6] National Institute for Health and Care Excellence. Obesity: identification, assessment and management. NICE guidelines CG189. November 2014. Available: http://www.nice.org.uk/guidance/cg189. Accessed 15 May 2015.

[7] Courcoulas AP, Goodpaster BH, Eagleton JK, Belle SH, Kalarchian MA, Lang W, et al Surgical vs medical treatments for type 2 diabetes mellitus: a randomized clinical trial. JAMA Surg. 2014;149:707–715. 10.1001/jamasurg.2014.467 24899268 PMC4106661

[8] O’Brien PE, Brennan L, Laurie C, Brown W. Intensive medical weight loss or laparoscopic adjustable gastric banding in the treatment of mild to moderate obesity: long-term follow-up of a prospective randomised trial. Obes Surg. 2013 23:1345–1353. 10.1007/s11695-013-0990-3 23760764

[9] Schauer PR, Kashyap SR, Wolski K, Brethauer SA, Kirwan JP, Pothier CE, et al Bariatric surgery versus intensive medical therapy in obese patients with diabetes. N Engl J Med. 2012;366:1567–1576. 10.1056/NEJMoa1200225 22449319 PMC3372918

[10] Mingrone G, Panunzi S, De Gaetano A, Guidone C, Iaconelli A, Leccesi L, et al Bariatric surgery versus conventional medical therapy for type 2 diabetes. N Engl J Med. 2012;366:1577–1585. 10.1056/NEJMoa1200111 22449317

[11] Campbell J, Dedman DJ, Eaton SC, Gallagher AM, Williams TJ. Is the CPRD GOLD population comparable to the U.K. population? Pharmacoepidemiol Drug Saf 2013;22(Suppl 1):280.

[12] Herrett E, Thomas SL, Schoonen WM, Smeeth L, Hall AJ. Validation and validity of diagnoses in the General Practice Research Database: a systematic review. Br J Clin Pharmacol. 2010;69:4–14. 10.1111/j.1365-2125.2009.03537.x 20078607 PMC2805870

[13] Bhaskaran K, Forbes H, Douglas I, Leon D, Smeeth L. Representativeness and optimal use of body mass index (BMI) in the UK Clinical Practice Research Datalink (CPRD). BMJ Open 2013;3:e003389 10.1136/bmjopen-2013-003389 24038008 PMC3773634

[14] Lewis JD, Bilker WB, Weinstein RB, Strom BL. The relationship between time since registration and measured incidence rates in the General Practice Research Database. Pharmacoepidemiol Drug Saf. 2005;14:443–451. 10.1002/pds.1115 15898131

[15] Austin PC. Balance diagnostics for comparing the distribution of baseline covariates between treatment groups in propensity-score matched samples. Stat Med. 2009;28:3083–3107. 10.1002/sim.3697 19757444 PMC3472075

[16] Stürmer T, Rothman KJ, Avorn J, Glynn RJ. Treatment effects in the presence of unmeasured confounding: dealing with observations in the tails of the propensity score distribution—a simulation study. Am J Epidemiol. 2010;172:843–854. 10.1093/aje/kwq198 20716704 PMC3025652

[17] Arterburn DE, Olsen MK, Smith V Livingston EH, Van Scoyoc L, Yancy WS Jr, et al Association between bariatric surgery and long-term survival. JAMA. 2015;313:62–70. 10.1001/jama.2014.16968 25562267

[18] Health and Social Care Information Centre. Health Survey for England 2013: health, social care and lifestyles. Summary of key findings. Available: http://www.hscic.gov.uk/catalogue/PUB16076/HSE2013-Sum-bklet.pdf. Accessed 20 May 2015.

[19] Still CD, Wood GC, Chu X, Manney C, Strodel W, Petrick A, et al Clinical factors associated with weight loss outcomes after Roux-en-Y gastric bypass surgery. Obesity. 2014;22:888–894. 10.1002/oby.20529 23804287 PMC3819407

[20] Angrisani L, Santonicola A, Iovino P, Formisano G, Buchwald H, Scopinaro NN. Bariatric surgery worldwide 2013. Obes Surg. 2015;25:1822–1832. 10.1007/s11695-015-1657-z 25835983

[21] Ogden CL, Carroll MD, Kit BK, Flegal KM. Prevalence of childhood and adult obesity in the United States, 2011–2012. JAMA. 2014;311:806–814. 10.1001/jama.2014.732 24570244 PMC4770258

[22] Wellbourn R, Small P, Finlay I, Sareela A, Somers S, Mahawar K, et al The United Kingdom National Bariatric Surgery Registry: second registry report. London: British Obesity & Metabolic Surgery Society; 2014.

[23] Canadian Agency for Drugs and Technologies in Health. Bariatric surgical procedures for obese and morbidly obese patients: a review of comparative clinical and cost-effectiveness, and guidelines. 24 4 2014 Ottawa: Canadian Agency for Drugs and Technologies in Health Available: http://www.ncbi.nlm.nih.gov/books/PMH0071568/. Accessed 20 November 2015. 25577938

[24] Zhang Y, Wang J, Sun X, Cao Z, Xu X, Liu D, et al Laparoscopic sleeve gastrectomy versus laparoscopic Roux-en-Y gastric bypass for morbid obesity and related comorbidities: a meta-analysis of 21 studies. Obes Surg. 2015;25:19–26. 10.1007/s11695-014-1385-9 25092167

[25] Peterli R, Borbély Y, Kern B, Gass M, Peters T, Thurnheer M, et al Early results of the Swiss Multicentre Bypass or Sleeve Study (SM-BOSS): a prospective randomized trial comparing laparoscopic sleeve gastrectomy and Roux-en-Y gastric bypass. Ann Surg. 2013;258:690–694. 10.1097/SLA.0b013e3182a67426 23989054 PMC3888472

[26] Young MT, Gebhart A, Phelan MJ, Nguyen NT. Use and outcomes of laparoscopic sleeve gastrectomy vs laparoscopic gastric bypass: analysis of the American College of Surgeons NSQIP. J Am Coll Surg. 2015;220:880–885. 10.1016/j.jamcollsurg.2015.01.059 25907869

[27] Manning S, Carter NC, Pucci A, Jones A, Elkalaawy M, Cheung WH, et al Age- and sex-specific effects on weight loss outcomes in a comparison of sleeve gastrectomy and Roux-en-Y gastric bypass: a retrospective cohort study. BMC Obes. 2014;1:12 10.1186/2052-9538-1-12 26217504 PMC4510900

[28] Goitein D, Raziel A, Szold A, Sakran N. Assessment of perioperative complications following primary bariatric surgery according to the Clavien-Dindo classification: comparison of sleeve gastrectomy and Roux-Y gastric bypass. Surg Endosc. 2015 4 11 E-pub ahead of print. 10.1007/s00464-015-4205-y 25861906

[29] Gloy VL, Briel M, Bhatt DL, Kashyap SR, Schauer PR, Mingrone G, et al Bariatric surgery versus non-surgical treatment for obesity: a systematic review and meta-analysis of randomised controlled trials. BMJ. 2013;347:f5934 10.1136/bmj.f5934 24149519 PMC3806364

[30] Sugerman HJ, Starkey JV, Birkenhauer R. A randomized prospective trial of gastric bypass versus vertical banded gastroplasty for morbid obesity and their effects on sweets versus non-sweets eaters. Ann Surg 1987;205:613–624. 3296971 10.1097/00000658-198706000-00002 PMC1493086

[31] Søvik TT, Taha O, Aasheim ET, Engström M, Kristinsson J, Björkman S, et al Randomized clinical trial of laparoscopic gastric bypass versus laparoscopic duodenal switch for superobesity. Br J Surg. 2010;97:160–166. 10.1002/bjs.6802 20035530

[32] Himpens J, Dapri G, Cadiere GB. A prospective randomized study between laparoscopic gastric banding and laparoscopic isolated sleeve gastrectomy: results after 1 and 3 years. Obes Surg. 2006;16:1450–1456. 17132410 10.1381/096089206778869933

[33] Dixon JB, O’Brien PE, Playfair J, Chapman L, Schachter LM, Skinner S, et al Adjustable gastric banding and conventional therapy for type 2 diabetes: a randomized controlled trial. JAMA. 2008;299:316–323. 10.1001/jama.299.3.316 18212316

[34] Picot J, Jones J, Colquitt JL, Gospodarevskaya E, Loveman E, Baxter L, et al The clinical effectiveness and cost-effectiveness of bariatric (weight loss) surgery for obesity: a systematic review and economic evaluation. Health Technol Assess. 2009;13:1–190,215–357,iii–iv. 10.3310/hta13410 19726018

[35] Lee WJ, Chong K, Ser KH, Lee YC, Chen SC, Chen JC, et al Gastric bypass vs sleeve gastrectomy for type 2 diabetes mellitus: a randomized controlled trial. Arch Surg. 2011;146:143–148. 10.1001/archsurg.2010.326 21339423

[36] Kehagias I, Karamanakos SN, Argentou M, Kalfarentzos F. Randomized clinical trial of laparoscopic Roux-en-Y gastric bypass versus laparoscopic sleeve gastrectomy for the management of patients with BMI < 50 kg/m2. Obes Surg. 2011;21:1650–1656. 10.1007/s11695-011-0479-x 21818647

[37] Karamanakos SN, Vagenas K, Kalfarentzos F, Alexandrides TK. Weight loss, appetite suppression, and changes in fasting and postprandial ghrelin and peptide-YY levels after Roux-en-Y gastric bypass and sleeve gastrectomy: a prospective, double blind study. Ann Surg 2008;247:401–407. 10.1097/SLA.0b013e318156f012 18376181

[38] Courcoulas AP, Christian NJ, Belle SH, Berk PD, Flum DR, Garcia L, et al Weight change and health outcomes at 3 years after bariatric surgery among individuals with severe obesity. JAMA. 2013;10:2416–2425. 10.1001/jama.2013.280928 PMC3955952 24189773

[39] Sjöström L. Review of the key results from the Swedish Obese Subjects (SOS) trial—a prospective controlled intervention study of bariatric surgery. J Intern Med. 2013;273:219–234. 10.1111/joim.12012 23163728

[40] Schauer PR, Bhatt DL, Kirwan JP, Wolski K, Brethauer SA, Navaneethan SD, et al Bariatric surgery versus intensive medical therapy for diabetes—3-year outcomes. N Engl J Med. 2014;370:2002–2013. 10.1056/NEJMoa1401329 24679060 PMC5451259

[41] Wentworth JM, Playfair J, Laurie C, Ritchie ME, Brown WA, Burton P, et al Multidisciplinary diabetes care with and without bariatric surgery in overweight people: a randomised controlled trial, Lancet Diabetes Endocrinol. 2014;2:545–552. 10.1016/S2213-8587(14)70066-X 24731535

[42] Svane MS, Madsbad S. Bariatric surgery—effects on obesity and related co-morbidities. Curr Diabetes Rev. 2014:10:208–214. 24934290 10.2174/1573399810666140616144141

[43] Sjöström L, Peltonen M, Jacobson P, Sjöström CD, Karason K, Wedel H, et al Bariatric surgery and long-term cardiovascular events. JAMA. 2012;307:56–65. 10.1001/jama.2011.1914 22215166

[44] Booth H, Khan O, Prevost T, Reddy M, Dregan A, Charlton J, et al Incidence of type 2 diabetes after bariatric surgery: population-based matched cohort study. Lancet Diabetes Endocrinol. 2014;2:963–968. 10.1016/S2213-8587(14)70214-1 25466723

[45] Sjöström L, Gummesson A, Sjöström CD, Narbro K, Peltonen M, Wedel H, et al Effects of bariatric surgery on cancer incidence in obese patients in Sweden (Swedish Obese Subjects Study): a prospective, controlled intervention trial. Lancet Oncol. 2009;10:653–662. 10.1016/S1470-2045(09)70159-7 19556163

[46] Bhaskaran K, Douglas I, Forbes H, dos-Santos-Silva I, Leon DA, Smeeth L. Body-mass index and risk of 22 specific cancers: a population-based cohort study of 5·24 million UK adults. Lancet. 2014;384:755–765. 10.1016/S0140-6736(14)60892-8 25129328 PMC4151483

