# Supplementary material for: Bariatric Surgery in the United Kingdom: A Cohort Study of Weight Loss and Clinical Outcomes in Routine Clinical Care
Source: PLoS Med. 2015 Dec 22;12(12):e1001925. doi: 10.1371/journal.pmed.1001925 (PMC4687869; doi:10.1371/journal.pmed.1001925)
Supplement: S1 Table — (DOCX) [file pmed.1001925.s001.docx]

**Supplementary Table 1: Rate of change in weight following surgery in people with type 2 diabetes stratified by surgery type**

| Time in Follow Up | Estimated weight change, kg/month (95% CI) |
| --- | --- |
| Type 2 diabetes + gastric band (n=425) |  |
| 1-4 months | -3.08 (-2.80 to -3.35) |
| 5-12 months | -0.78 (-0.63 to -0.93) |
| 13-48 months | 0.02 (-0.06 to 0.10) |
| Type 2 Diabetes + gastric bypass (n=652) |  |
| 1-4 months | -6.75 (-6.57 to -6.94) |
| 5-12 months | -1.32 (-1.21 to -1.44) |
| 13-48 months | 0.05 (-0.03 to 0.13) |
| Type 2 diabetes + sleeve gastrectomy (n=255) |  |
| 1-4 months | -6.32 (-5.99 to -6.65) |
| 5-12 months | -0.95 (-0.74 to -1.16) |
| 13-48 months | 0.21 (0.06 to 0.35) |
