# Supplementary material for: Bariatric Surgery in the United Kingdom: A Cohort Study of Weight Loss and Clinical Outcomes in Routine Clinical Care
Source: PLoS Med. 2015 Dec 22;12(12):e1001925. doi: 10.1371/journal.pmed.1001925 (PMC4687869; doi:10.1371/journal.pmed.1001925)
Supplement: S2 Table — (DOCX) [file pmed.1001925.s002.docx]

**Supplementary Table 2 Association between bariatric surgery and health outcomes without trimming patients with extreme propensity scores**

| Outcome | Exposure | N | Number with outcome | Median FU (y) | Hazard Ratio (95% confidence interval) |
| --- | --- | --- | --- | --- | --- |
| Type 2 diabetes  First diag  First OAD  First insulin  Resolution | No surgery  Surgery  No surgery  Surgery  No surgery  Surgery  No surgery  Surgery | 2,586  2,560  3,196  3,055  3,626  3,628  1,247  1,115 | 239 (9.2)  166 (6.5)  153 (4.8)  39 (1.3)  48 (1.3)  11 (0.3)  54 (4.3)  321 (28.8) | 2.9  3.1  2.9  3.2  3.0  3.1  2.6  1.8 | -  0.67 (0.55-0.81)  -  0.26 (0.18-0.36)  -  0.23 (0.12-0.44)  -  8.57 (6.42-11.45) |
| Hypertension  Diagnosis  Resolution | No surgery  Surgery  No surgery  Surgery | 2,559  2,567  1,303  1,296 | 223 (8.7)  85 (3.3)  8 (0.6)  48 (3.7) | 2.8  2.9  2.8  2.8 | -  0.36 (0.28-0.46)  -  5.98 (2.82-12.67) |
| Angina | No surgery  Surgery | 3,663  3,619 | 70 (1.9)  42 (1.2) | 3.0  3.0 | -  0.59 (0.40-0.86) |
| MI | No surgery  Surgery | 3,838  3,847 | 18 (0.5)  5 (0.1) | 3.0  3.1 | -  0.27 (0.10-0.72) |
| Stroke | No surgery  Surgery | 3,855  3,846 | 20 (0.5)  19 (0.5) | 3.0  3.0 | -  0.94 (0.50-1.77) |
| Fractures  Hip  Wrist  Spine  Any | No surgery  Surgery  No surgery  Surgery  No surgery  Surgery  No surgery  Surgery | 3,855  3,847  3,566  3,486  3,861  3,857  3,636  3,596 | 8 (0.2)  8 (0.2)  18 (0.5)  30 (0.8)  11 (0.3)  16 (0.4)  33 (0.9)  42 (1.2) | 3.0  3.1  3.0  3.0  3.0  3.0  3.0  3.0 | -  0.98 (0.37-2.62)  -  1.69 (0.94-3.03)  -  1.46 (0.68-3.14)  -  1.28 (0.81-2.02) |
| Fatty liver | No surgery  Surgery | 3,832  3,825 | 26 (0.6)  13 (0.3) | 3.0  3.1 | -  0.49 (0.25-0.96) |
| Obstructive sleep apnoea | No surgery  Surgery | 3,742  3,406 | 77 (2.1)  39 (1.2) | 3.0  3.1 | -  0.54 (0.37-0.79) |
| Any cancer | No surgery  Surgery | 3,640  3,603 | 142 (3.9)  130 (3.6) | 2.9  3.0 | -  0.91 (0.72-1.16) |
| Mortality | No surgery  Surgery | 3,882  3,882 | 59 (1.5)  52 (1.3) | 3.0  3.1 | -  0.88 (0.60-1.27) |
