# Supplementary material for: Bariatric Surgery in the United Kingdom: A Cohort Study of Weight Loss and Clinical Outcomes in Routine Clinical Care
Source: PLoS Med. 2015 Dec 22;12(12):e1001925. doi: 10.1371/journal.pmed.1001925 (PMC4687869; doi:10.1371/journal.pmed.1001925)
Supplement: S3 Table — (DOCX) [file pmed.1001925.s003.docx]

**Supplementary Table 3. Association between bariatric surgery and health outcomes stratified by surgery subtype, baseline type 2 diabetes and baseline CVD**

| Outcome | Exposure | Hazard Ratio, 95% CI | | | | |
| --- | --- | --- | --- | --- | --- | --- |
|  |  | People with CVD | People with diabetes | Gastric band | Gastric bypass | Sleeve gastrectomy |
| Type 2 diabetes  First diag  First OAD  First insulin  Resolution | No surgery  Surgery  No surgery  Surgery  No surgery  Surgery  No surgery  Surgery | 0.61 (0.44-0.87)  0.25 (0.13-0.48)  -  0.23 (0.10-0.57)  -  7.53 (5.13-11.06) | N/A | -  0.64 (0.50-0.81)  -  0.28 (0.18-0.43)  -  0.29 (0.14-0.62)  -  1.9 (1.4-2.6) | -  1.02 (0.66-1.59)  -  0.33 (0.13-0.81)  -  0.18 (0.03-1.34)  -  8.2 (5.8-11.5) | -  0.62 (0.43-0.89)  -  0.17 (0.07-0.38)  -  0.08 (0.01-0.56)  -  10.95 (8.41-14.26) |
| Hypertension  Diagnosis  Resolution | No surgery  Surgery  No surgery  Surgery | -  0.18 (0.04-0.91)  -  N/A | -  0.33 (0.21-0.53)  -  5.73 (1.68-19.57) | -  0.41 (0.31-0.55)  -  1.86 (0.82-4.21) | -  0.33 (0.16-0.67)  -  8.86 (3.76-20.91) | -  0.20 (0.11-0.36)  -  8.23 (4.05-16.75) |
| Angina | No surgery  Surgery | -  0.50 (0.29-0.85) | -  0.45 (0.24-0.85) | -  0.74 (0.48-1.14) | -  0.70 (0.30-1.63) | -  0.26 (0.10-0.64) |
| MI | No surgery  Surgery | -  0.30 (0.10-0.93) | -  0.38 (0.12-1.19) | -  0.29 (0.08-0.98) | -  N/A | -  0.39 (0.09-1.67) |
| Stroke | No surgery  Surgery | -  1.03 (0.43-2.47) | -  0.57 (0.24-1.36) | -  0.67 (0.28-1.59) | -  0.75 (0.17-3.26) | -  1.49 (0.65-3.43) |
| Fractures  Hip  Wrist  Spine  Any | No surgery  Surgery  No surgery  Surgery  No surgery  Surgery  No surgery  Surgery | -  1.50 (0.25-9.00)  -  2.83 (1.09-7.32)  -  1.75 (0.63-4.83)  -  2.00 (0.95-4.19) | -  1.65 (0.27-9.92)  -  1.57 (0.66-3.71)  -  1.31 (0.47-3.62)  -  1.17 (0.57-2.38) | -  1.24 (0.39-3.90)  -  1.79 (0.93-3.44)  -  0.98 (0.36-2.67)  -  1.33 (0.78-2.26) | -  2.28 (0.47-11.05)  -  0.49 (0.06-3.66)  -  0.74 (0.09-5.72)  -  0.81 (0.25-2.66) | -  0.50 (0.06-4.10)  -  1.57 (0.68-3.62)  -  2.21 (0.85-5.72)  -  1.10 (0.54-2.25) |
| Fatty liver | No surgery  Surgery | -  0.65 (0.23-1.83) | -  0.81 (0.29-2.23) | -  0.62 (0.29-1.33) | -  0.74 (0.18-3.15) | -  0.26 (0.06-1.12) |
| Obstructive sleep apnoea | No surgery  Surgery | -  0.58 (0.32-1.07) | -  0.67 (0.34-1.34) | -  0.68 (0.44-1.05) | -  0.32 (0.08-1.29) | -  0.35 (0.15-0.80) |
| Any cancer | No surgery  Surgery | -  0.80 (0.55-1.18) | -  0.80 (0.53-1.20) | -  0.85 (0.63-1.14) | -  1.17 (0.72-1.90) | -  1.06 (0.75-1.49) |
| Mortality | No surgery  Surgery | -  0.86 (0.53-1.41) | -  1.00 (0.60-1.67) | -  0.57 (0.32-0.99) | -  1.50 (0.79-2.85) | -  1.33 (0.79-2.22) |
