# Supplementary material for: Bariatric Surgery in the United Kingdom: A Cohort Study of Weight Loss and Clinical Outcomes in Routine Clinical Care
Source: PLoS Med. 2015 Dec 22;12(12):e1001925. doi: 10.1371/journal.pmed.1001925 (PMC4687869; doi:10.1371/journal.pmed.1001925)
Supplement: S1 Text — (DOCX) [file pmed.1001925.s004.docx]

**Supplementary Material: CPRD Code list for Bariatric Surgery**

*medcode* *description*

18863 Laparoscopic adjustable gastric banding

56926 Partitioning of stomach using staples

59622 Mason vertical banded gastroplasty

88474 Partitioning of stomach using band

89148 Laparoscopic sleeve gastrectomy

89259 Sleeve gastrectomy NEC

90517 Duodenal switch

90600 Partitioning of stomach NEC

93378 Sleeve gastrectomy and duodenal switch

95929 Laparoscopic gastric bypass
