# Supplementary material for: Bariatric Surgery in the United Kingdom: A Cohort Study of Weight Loss and Clinical Outcomes in Routine Clinical Care
Source: PLoS Med. 2015 Dec 22;12(12):e1001925. doi: 10.1371/journal.pmed.1001925 (PMC4687869; doi:10.1371/journal.pmed.1001925)
Supplement: S3 Text — (DOC) [file pmed.1001925.s006.doc]

**Measuring the long term outcomes of bariatric surgery – a UK population-based cohort study**

Dr Ian Douglas

Department of Epidemiology and Population Health

London School of Hygiene and Tropical Medicine

Keppel Street, London WC1E 7HT

E mail: ian.douglas@lshtm.ac.uk

Tel: 020 7927 2968

Fax: 020 7580 6897

Professor Liam Smeeth

Department of Epidemiology and Population Health

London School of Hygiene and Tropical Medicine

Keppel Street, London WC1E 7HT

E mail: liam.smeeth@lshtm.ac.uk

Tel: 020 7927 2296

Fax: 020 7580 6897

**Lay Summary of Research**

Obesity is becoming more common in the UK and already affects ~24% of the population. Common health problems associated with obesity include heart disease, diabetes and mobility problems, all of which can significantly impact on the quality of life of those affected. Surgical and drug treatments for obesity are now being used more frequently, and there is little evidence comparing one treatment with another and very little is know about the long term effects of treatment (e.g. over several years). We aim to combine two UK primary care databases – the GPRD and QRESEARCH, and conduct a cohort study to assess the effect of treatments on a range of outcomes including mortality, post-operative complications, type 2 diabetes, cardiovascular disease, fractures, cancer, fatty liver, acute liver injury, obstructive sleep apnoea, weight, body mass index and blood lipid profile. We will compare the occurrence of these outcomes in obese people who receive weight loss surgery, obese people who receive weight loss medication, and obese people who receive no treatment. The results of this study will improve the evidence base available to doctors and patients trying to decide the best treatment options for people with obesity.

**Objectives**

To measure long term major clinical outcomes in people who have had bariatric surgery and compare them with a) people receiving pharmaceutical treatment for obesity and b) obese people receiving no treatment.

**Specific Aims**

Rates of the following outcomes will be compared between groups: Mortality, post-operative complications, type 2 diabetes, cardiovascular disease, fractures, cancer, fatty liver, acute liver injury and obstructive sleep apnoea. Changes over time will be compared against baseline and between groups for the following outcomes: weight, body mass index (BMI), lipid profile.

**Background & Rationale**

The prevalence of obesity (BMI>=30) in the UK is currently ~24% and will almost certainly increase over the next five to ten years (Craig, 2006). As a consequence, obesity related health problems such as type-2 diabetes, cardiovascular disease and mobility problems are also common. Combined with psychological stress and social stigma, obesity has a substantial impact on individual quality of life and clinical outcomes (Throsby, 2007).

Various approaches are available to tackle obesity, including diet and exercise, but in many cases surgical or pharmacological treatments may also be used. Randomised trials of surgical and pharmacological interventions for obesity have generally been small and short term, since trials are usually powered to demonstrate efficacy, whilst the reporting of safety outcomes is often incomplete or inconsistent (Papanikolaou 2006, Black 1996). The “real world” safety and effectiveness of these interventions as used in UK secondary/primary care has not been well studied over long periods.

Hospital Episode Statistics were used by Burns et al to evaluate short-term mortality and readmission rates after bariatric surgery in England, comparing gastric banding with bypass in ~7,000 patients (Burns et al, 2010). However, longer term effects and weight loss were not assessed and comparisons with non-surgical treatments were unavailable.

A systematic review of the clinical effectiveness and cost effectiveness of bariatric surgery for obesity was published as an HTA report in 2009 (Picot et al., 2009). One of the studies included, the Swedish Obese Subjects (SOS) prospective cohort study, has been reporting long term outcomes such as weight loss, typical obesity-related clinical outcomes and QoL for up to 10 years in ~2,000 subjects undergoing bariatric surgery compared with a similar number of obese subjects not receiving surgery. They report QoL peaking at 12 months post-surgery, with a subsequent decline and stabilisation at levels above baseline up to 10 years later; a reduction in incident diabetes, hypertriglyceridemia, hyperuricaemia; resolution of type 2 diabetes, hypertension, hypertriglyceridemia and hyperuricaemia; and no difference in incident hypertension or hypercholesterolaemia resolution when comparing surgical with non-surgical treatment (Karlsson et al., 2007).

A further study of the Swedish population compared >1,000 patients receiving bariatric surgery with >5,000 obese non-surgery patients; surgery was followed by a decreased incidence of type 2 diabetes, hyperlipidemia and mortality but no difference for hypertension, angina, MI or stroke (Marsk et al, 2010). Quality of life and weight assessments were not included in this report.

Other relevant studies in non-UK settings have mostly involved small patient numbers, measured a limited range of outcomes or had poor follow up rates, making findings difficult to interpret (Batsis et al, 2010; Picot et al, 2009).

Large scale, comprehensive population-based research measuring the impact of surgery for obesity in the UK, in comparison with other forms of care is therefore lacking. We believe we are ideally placed to answer a number of important questions that will help inform future treatment for obesity.

**Study Type**

This is a hypothesis testing study; throughout the null hypothesis will be that there is no clinical difference in outcomes following bariatric surgery or pharmacological treatment for obesity, compared with receiving no treatment.

**Study Design and Study Population**

A combination of two UK databases offers an excellent opportunity to study the clinical effects of interventions for obesity; the UK General Practice Research Database (GPRD) and QRESEARCH. In combination the GPRD and QRESEARCH contain information from >1100 UK general practices, and have current and historical data for >20 million patients. Continuous patient records include details of all consultations, diagnoses, prescribed medicines and basic demographic data. Patients are representative of the England & Wales population in terms of age, sex and geographical distribution [refs]. Data quality is subject to rigorous checks and audits and >500 peer reviewed studies have been published using GPRD or QRESEARCH data including studies investigating unintended effects of medication (GPRD, 2010; QRESEARCH, 2010). GPRD and QRESEARCH draw information from mutually exclusive practices and are therefore fully complementary.

*3.1 Study Design - Patient population, exposures of interest, inclusion/exclusion criteria*

The cohorts will comprise obese patients registered in the GPRD and QRESEARCH:

- Eligible patients will be those aged 18+ years with at least 12 months research standard registration time in the GPRD or QRESEARCH AND with one of the following exposures recorded by the study start:
  - A record of bariatric surgery with at least 12 months prior registration to ensure incident status (specific type of bariatric surgery used will be determined using data recorded in patient records); OR
  - A first prescription for orlistat with at least 12 months prior registration to ensure incident usage; OR:
  - A record of BMI>30 with neither bariatric surgery nor a prescription for orlistat – these patients will act as the **comparison group**
- The date of surgery or first orlistat prescription will be termed the index date. Each patient with surgery or orlistat will be matched with up to 5 obese patients without a record of these treatments, on age (within 5 years), sex and practice with at least 12 months research standard registration prior to the relevant index date.
- Patients will be followed until the earliest of; an outcome of interest, death, transfer out of the practice or last data collection date.
- As a secondary analysis we will also be able to assess exposure to sibutramine. Whilst the marketing authorisation for sibutramine has been suspended, there is uncertainty over whether marketing may be resumed in the future, and the effects of this drug are therefore still of broad interest.

**Sample Size/Power Calculation**

Counts received from the GPRD show:

- ~2,000 registered patients have had bariatric surgery, with a mean follow up of 2.4 years
- ~80,000 patients have received a prescription for orlistat
- >1.4 million patients have a BMI >=30

QRESEARCH contains data on a similar number of patients to GPRD and we therefore anticipate the number of eligible patients for this study would be roughly double the numbers shown above.

Assuming we match 5 untreated patients to each patient with an obesity intervention, we would have 80% power to detect a reduction in mortality over 4 years amongst those undergoing surgery from 5% to 3.9% (over the whole 4 year period).

**Exposures**

Exposures will be defined as follows:

- First record of bariatric surgery with at least 12 months prior UTS registration
- A first prescription for orlistat with at least 12 months prior registration to ensure incident usage
- A first prescription for sibutramine with at least 12 months prior registration to ensure incident usage

**Outcomes**

Outcomes have been chosen as events that are well recorded by GPs as part of routine patient care, regardless of where a diagnosis is made (by the GP or in secondary/tertiary centres). Numerous studies have been published looking at these outcomes using data from the GPRD/QRESEARCH and validity has been shown to be high (GPRD, 2010; QRESEARCH, 2010). The following outcomes will therefore be ascertained for all patients in the cohort:

- Death
- Change in Weight
- Change in BMI
- Diabetes
  - First diagnosis
  - Progression to monotherapy with oral antidiabetic agent
  - Progression to polytherapy with oral antidiabetic agents
  - Progression to insulin therapy
  - Resolution (noted in GP record as patient removal from diabetes QOF register)
- Cardiovascular disease
  - Incident MI
  - Incident stroke
  - Onset of hypertension
  - Onset of angina
  - Improvement of hypertension determined by permanently stopping antihypertensive medication, i.e. at least 6 months without a further prescription
- Osteoporotic Fractures (hip, spine, wrist)
- Acute liver injury as indicated by a diagnostic code or a record of alanine transferase at >3X upper limit of normal in patients without chronic liver disease.
- Fatty liver
- Incident cancer diagnoses
- Obstructive sleep apnoea
- Change in lipid profile

In addition, within the GPRD, Hospital Episode Statistics (HES) data will be available for a large proportion of the cohort. We will therefore be able ascertain post-operative outcomes, including post-operative haemorrhage, post-operative infection, ITU admission, re-admission to hospital and re-operation. Also, within the GPRD, detailed information on acute coronary events will be available via linkages with the Myocardial Ischaemia National Audit Project (MINAP).

The research team has considerable experience in developing algorithms for determining outcomes based on both GPRD and is developing expertise with HES data – algorithms for specific outcomes are available on request.

**Statistical Analysis and Covariates**

*Baseline Comparisons*

We will use baseline data to compare the background characteristics of patients receiving surgical and pharmacological interventions for obesity with those receiving no intervention. This will give a broad picture of how these interventions are being targeted in the UK as a whole. Odds ratios and 95% confidence intervals will allow us to determine statistically and clinically relevant differences, using patients with no intervention as the baseline group. As a subgroup analysis, differences in the targeting of specific forms of bariatric surgery will also be determined. **Covariates** to be assessed will include age, sex, weight, BMI, smoking status, alcohol consumption and a range of co-morbidities such as coronary heart disease, hypertension and diabetes.

*Cohort analysis*

We will compare outcomes between patients receiving surgical and pharmacological interventions for obesity with those receiving no intervention. It is anticipated that these three groups of patients will differ in fundamental ways related to health outcomes that could represent important bias and confounding in this study. For this reason we will calculate propensity scores for each patient, capturing their likelihood to receive either surgery or a prescribed anti-obesity therapy. Using the propensity scores we will ensure comparisons are only made between patients with similar background characteristics (Sturmer et al, 2010). Cox proportional hazard regression will be used to conduct time to event analyses for death, diabetes, cardiovascular disease, fractures and liver disease, adjusting for the propensity score. Patients with no intervention will form the baseline group.

In addition, anonymised, linked Hospital Episode Statistic (HES) data are available for patients in the GPRD and will allow us to look in detail at hospital records for patients who undergo bariatric surgery (e.g. type of surgery, any complication that arise).

Patients who receive orlistat and later have surgery will move from one exposure group to the other, according to current treatment. We anticipate that very few patients will receive orlistat following surgery and any patients who appear to do so will be excluded from the analysis.

For exposure to orlistat, follow up time will be censored 3 months after the date when prescribing information indicates treatment was stopped. This period allows for a degree of medication stockpiling and a treatment washout period. Similarly, follow up time for controls matched to orlistat recipients will be censored at the same date ensuring similar follow up in comparison groups.

Sensitivity analyses will stratify orlistat exposure on dose and duration using recorded prescribing information to estimate daily dose and duration of treatment.

Where possible, we will calculate number needed to treat (NNT) or number needed to harm (NNH) for clinical outcomes where a treatment effect is seen.

Linear regression will be used to compare changes in weight and BMI between surgical or pharmacological interventions and no intervention, adjusting for the propensity score. Changes will be calculated annually.

Similarly, a paired t-test will be used within each treatment group to determine changes in weight and BMI from baseline (pre-intervention). Changes will be calculated annually.

Absolute rates will also be determined for post-operative complications (post-operative haemorrhage, post-operative infection, ITU admission, re-admission to hospital and re-operation). Cox regression will be used to compare the hazard for each event between different types of bariatric surgery.

Initially the analysis will be done separately for QRESEARCH and GPRD data. It is anticipated the results will be very similar since the two datasets represent different samples of the same underlying population. Assuming the results are similar, data will be pooled, and a single analysis will be done, which will increase precision.

**Patient/User Group Involvement**

We do not believe this research would benefit from patient group involvement at this stage, but it is possible that future research may well benefit from such involvement.

**Limitations**

As mentioned above, it is likely that patients receiving surgery, pharmacological treatment or no intervention are likely to differ substantially. We will attempt to account for this by using propensity score matching, though we acknowledge that this may have an impact on power if few matches are available. Until the data are available we cannot predict the extent of this limitation.

Some patients may pay for their own surgical treatment abroad, which may not be instantly recorded by their GP, or in extreme cases may not be recorded at all. This could lead to a misclassification of exposure in the non-treatment group, though it is anticipated this will only affect a minority of this group.

Similarly orlistat is available over the counter and such use will not be well captured in primary care data. We will therefore conduct a sensitivity analysis of the effects of orlistat, censoring at the time the license first allowed over the counter sales (early 2009)

Data are likely to be missing for a small number of patients on some variables (e.g. smoking, alcohol consumption and BMI). For smoking and alcohol, missing values will be treated as a separate stratum during the construction of the propensity score. For BMI, we anticipate mostly complete recording as it is fundamental to the care of this patient group. However, in rare cases of missing post-index BMI measures, these patients will be excluded from the analysis for changes in BMI.

**Plans for disseminating and communicating study results**

We intend to publish our findings in a peer reviewed journal and also to present them at relevant scientific conferences**.**

**Amendment**

A number of amendments to this protocol are now sought:

1. Due to funding constraints, it will not be possible to access QRESEARCH data and the study will be restricted to CPRD data only. We do not anticipate major changes to the feasibility of the study, as the current number of patients in the CPRD with bariatric surgery is now >3,000.
2. Following initial data processing it became apparent that matches for bariatric surgery patients, drawn from the population of patients with a BMI >30 were very dissimilar from the surgery patients, mainly because the surgery group tended to have a much higher BMI. Since the guidelines for surgery include a requirement of BMI >=40 in most instances, we propose matches to be drawn from a pool of patients with any measure of BMI >=40 during their registration.
3. For the analysis of weight loss, we intend to measure the effects to a maximum of three years post-intervention, largely driven by the amount of follow up available for surgery patients
4. Following statistical advice from Krishnan Bhaskaran, it was decided that mixed effects linear regression should be used to assess changes in weight and BMI over time within each group. We anticipate a non-uniform rate of change over follow up and will model this using splines. The Akaike Information Criterion (AIC) will be used to determine optimal spline knot points. From the final model, weight/BMI and 95% confidence intervals for the study population will be estimated. The rate of change in weight or BMI and 95% confidence intervals will be calculated for each spline component.
5. For all other outcomes, orlistat patients will be censored at the end of follow up, analogous to an intention to treat type analysis, so the long term consequences of the decision to start a course of treatment with orlistat can be measured, regardless of subsequent treatment. As a sensitivity analysis, follow up will be censored at the estimated date treatment was stopped, based on prescribing records.
6. Subgroup analyses according to underlying diabetes and cardiovascular disease status will be carried out, in line with NICE recommendations for research on treatments for obesity. Additionally, where numbers permit, analyses of bariatric surgery patients will be stratified by surgery type.
7. As a clarification, patients who have bariatric surgery and later receive orlistat will be excluded from orlistat analyses only.
8. We intend to divide the study into two separate research papers; one addressing changes in weight and BMI, and the other addressing all other outcomes.
9. **ADDITIONAL AMENDMENT 22 MAY 2014:** We plan to compare the risk of cardiovascular events in patients prescribed sibutramine with those prescribed orlistat. Sibutramine marketing was suspended in 2010 due to an increased risk of adverse cardiovascular outcomes. A review by the European Medicines Agency, found an “increased risk of non­fatal myocardial infarction and stroke, which outweighed the possible benefits of the medication through weight­loss” (Williams, 2010)[2]. This risk was clarified by the Sibutramine Cardiovascular Outcomes Trial (SCOUT; James et al, 2010), which examined cardiovascular outcomes in those at increased risk of cardiovascular events (i.e. individuals with diabetes mellitus, pre­existing cardiovascular disease or both). SCOUT randomized over 10,000 patients to either sibutramine or placebo. The primary outcome was time from randomization to non­fatal myocardial infarction, non­fatal stroke, resuscitation after cardiac arrest, and cardiovascular death. The risk of a primary outcome event was increased by 16% in the sibutramine group compared with placebo (hazard ratio, 1.16; 95% confidence interval [CI], 1.03 to 1.31; P=0.02), with overall incidences of 11.4% and 10.0%, in the two groups, respectively. This increase in primary outcome events was made up of non­fatal cardiovascular events, rather than cardiovascular death (hazard ratio, 0.99; 95% CI, 0.82 to 1.19; P=0.90). Also the increased risk was only shown in the groups with pre­existing cardiovascular disease (who previously had been excluded in prescribing guidance).

Orlistat remains the only prescribed drug for weight loss in the EU, and to date, no direct comparison has been made between orlistat and sibutramine regarding the risk of vascular outcomes. We will directly compare the risk of myocardial infarction and all cause mortality in patients prescribed orlistat and sibutramine. These outcomes are already included in this study, but the direct comparison between orlistat and sibutramine was not previously specified. This work will be done as part of an Epidemiology MSc summer project, by Joseph Hayes. Specifically we will:

- 1. determine the relative risk of myocardial infarction (both fatal and non­fatal) in patients prescribed sibutramine or orlistat
  2. determine the relative risk of myocardial infarction in high­risk patients (i.e with cardiovascular risk factors) prescribed sibutramine or orlistat
  3. determine time to all­cause mortality in patients prescribed sibutramine or orlistat

1. Analysis plan

Initially, basic descriptive analyses of event rates will be conducted in each treatment group. Cox proportional hazard models will be used to investigate univariable and multivariable hazard ratios for the outcomes. Adjustment will be made for potential confounders (covariates to be assessed will include age, sex, weight, BMI, smoking status, alcohol consumption and a range of co-morbidities such as coronary heart disease, hypertension and diabetes). Following traditional regression analysis, propensity scores will be calculated to further explore possible prescribing biases and confounding, based on patient status with respect to the covariates listed above, and their orlistat/sibutramine exposure status. Our primary intention is to match sibutramine and orlistat patients with similar propensity scores, excluding any patients with scores in non-overlapping regions of the score distribution. Cox proportional hazards models will then be used to calculate Hazard Ratios for each outcome. Sensitivity analyses will also be used to estimate how large an effect an unknown confounder would need to have to eliminate or bring about any observed effect. We will also determine whether any effect is modified by age, sex, and area level social deprivation.

The cohort of obese patients already extracted contains over 27,000 patients prescribed sibutramine and 90,000 patients prescribed orlistat. Assuming a baseline 7% annual event rate (a two sided type I error rate of 0.05, and power set at 90%) to detect a 10% difference in hazard ratio between orlistat and sibutramine would require a sample size of 19,000 patients prescribed sibutramine and 58,000 patients prescribed orlistat. Assuming orlistat is not associated with an increased risk of vascular events, we estimate we have adequate power to detect an increased risk of MI, similar to the increased risk of MI and stroke detected in the SCOUT trial.

**Amendment 21 May 2015**

The following amendment is proposed

1. Health Survey for England estimates that currently 1.4 million residents in England are morbidly obese (BMI > 40kg/m2 ). For all outcomes where surgery appeared to have an effect, we will estimate the absolute number of outcomes that surgery could potentially prevent, by applying the relative risks obtained in our analysis to the rate of events detected in the non-surgery group, and scaling to 1.4 million people.
2. In order to maximise the study population size, we will no longer use MINAP and HES to ascertain MIs and will instead use first recorded MI in the CPRD GOLD record.

**References**

BATSIS, J. A., LOPEZ-JIMENEZ, F., COLLAZO-CLAVELL, M. L., CLARK, M. M., SOMERS, V. K. & SARR, M. G. 2009. Quality of life after bariatric surgery: a population-based cohort study. Am J Med, 122, 1055 e1-1055 e10.

BLACK, N. 1996. Why we need observational studies to evaluate the effectiveness of health care. BMJ, 312, 1215-8.

BURNS, E. M., NASEEM, H., BOTTLE, A., LAZZARINO, A. I., AYLIN, P., DARZI, A., MOORTHY, K. & FAIZ, O. 2010. Introduction of laparoscopic bariatric surgery in England: observational population cohort study. BMJ, 341, c4296.

CRAIG, R. M., J 2008. Health Survey for England 2006 Latest Trends. In: HEALTH, D. O. (ed.). The Information Centre.

GPRD. 2010. Bibliography [Online]. Available: http://gprd.com/bibliography/ [Accessed].

Health Survey for England 2013 Health, social care and lifestyles Summary of key findings; The Health and Social Care Information Centre. Available from http://www.hscic.gov.uk/catalogue/PUB16076/HSE2013-Sum-bklet.pdf (Accessed 20 May 2015

KARLSSON, J., TAFT, C., RYDEN, A., SJOSTROM, L. & SULLIVAN, M. 2007. Ten-year trends in health-related quality of life after surgical and conventional treatment for severe obesity: the SOS intervention study. Int J Obes (Lond), 31, 1248-61.

MARSK, R., NASLUND, E., FREEDMAN, J., TYNELIUS, P. & RASMUSSEN, F. 2010. Bariatric surgery reduces mortality in Swedish men. Br J Surg, 97, 877-83.

PAPANIKOLAOU, P. N., CHRISTIDI, G. D. & IOANNIDIS, J. P. 2006. Comparison of evidence on harms of medical interventions in randomized and nonrandomized studies. CMAJ, 174, 635-41.

PICOT, J., JONES, J., COLQUITT, J. L., GOSPODAREVSKAYA, E., LOVEMAN, E., BAXTER, L. & CLEGG, A. J. 2009. The clinical effectiveness and cost-effectiveness of bariatric (weight loss) surgery for obesity: a systematic review and economic evaluation. Health Technol Assess, 13, 1-190, 215-357, iii-iv.

QRESEARCH. 2010. Publications (2010). Available at http://www.qresearch.org/SitePages/publications.aspx [Online]. [Accessed].

STURMER, T., ROTHMAN, K. J., AVORN, J. & GLYNN, R. J. 2010. Treatment effects in the presence of unmeasured confounding: dealing with observations in the tails of the propensity score distribution--a simulation study. Am J Epidemiol, 172, 843-54.

THROSBY, K. 2007. "How could you let yourself get like that?" Stories of the origins of obesity in accounts of weight loss surgery. Soc Sci Med, 65, 1561-71.

Williams G (2010) Withdrawal of sibutramine in Europe. BMJ 340.

James WPT, Caterson ID, Coutinho W, Finer N, Van Gaal LF, et al. (2010) Effect of Sibutramine on Cardiovascular Outcomes in Overweight and Obese Subjects. New England Journal of Medicine 363: 905­917
